# 3-year overall survival benefit of systematic follow-up with 18F-FDG PET/CT in asymptomatic patients treated for head and neck squamous cell carcinoma: a multicenter study

**DOI:** 10.1007/s00259-025-07147-9

**Published:** 2025-02-26

**Authors:** C. Mahéo, R. Abgral, C. Clément, O. Malard, F. Espitalier, C. Ferron, O. Delcroix, R. Le Pennec, U. Schick, V. Tissot, G. Le Gal, F. Kraeber-Bodéré, T. Eugène, R. Marianowski-, P. Y. Salaün, Jean-Christophe Leclère-

**Affiliations:** 1 Department of Head and Neck Surgery, CHU de Brest, Brest, 29200 France; 2 Department of Nuclear Medicine, Brest University Hospital (CHU de Brest), Brest, 29200 France; 3 UMR Inserm 1304 GETBO, Univ Brest, Brest, 29200 France; 4 Department of Head and Neck Surgery, CHU de Nantes, Nantes, 44000 France; 5 Department of Radiotherapy, CHU de Brest, Brest, 29200 France; 6 Department of Radiology, CHU de Brest, Brest, 29200 France; 7 Clinical Trials Centre CIC 1412, CHU de Brest, Brest, 29200 France; 8 Department of Nuclear Medicine, CHU de Nantes, Nantes, 44000 France; 9 Laboratoire LIEN, Univ Brest, Brest, 29200 France

**Keywords:** HNSCC, PET/CT, Follow-up, Overall survival

## Abstract

**Purpose:**

Patients diagnosed with head and neck squamous cell carcinoma (HNSCC) face a significant risk of locoregional recurrence within the first two years after treatment. While early detection of recurrence could potentially improve patient outcomes, the impact of such detection on survival remains uncertain. The aim was to assess the potential benefit of a systematic post-treatment follow-up strategy using 18 F-FDG PET/CT imaging on overall survival.

**Methods:**

In this multicenter case-control study, patients were treated in two health areas from two different regions in France. All adults diagnosed with histologically confirmed HNSCC and treated between January 2017 and December 2020 with curative intent, with a complete response on imaging were included in the study. Primary endpoint was 3-year overall survival. The log-rank test was used to compare 3-year OS rates between the CFU (conventional follow-up) and PET/CT groups. A Cox regression model was used to assess the effect of the addition of 18 F-FDG PET/CT on survival outcomes.

**Results:**

A total of 697 patients were included (534 males [77%], median age[IQR] 62[57–69] years); 508 patients had CFU and 189 patients had CFU + systematic annual 18 F-FDG PET/CT. Cox regression analysis showed a protective effect (OR = 0.56, 95%CI:0.397–0.795, *p* = 0.001) of systematic 18 F-FDG PET/CT. The 3-year OS in the PET/CT group was better than in the CFU group (83.5 ± 2.8% vs. 73.4 ± 2.1%, *p* = 0.008). The analysis based on stage showed a significantly better 3-year OS for advanced stage III/IV in the PET/CT group (*n* = 124) than in the CFU group (*n* = 312)(79.9 ± 3.7% vs. 71.5 ± 2.7%, *p* = 0.045) as well as for early stage I/II (90.5 ± 3.7% vs. 76.3 ± 3.2%, *p* = 0.047).

**Conclusion:**

In this multicenter study, the use of 18 F-FDG PET/CT as an alternative to annual chest CT in the follow-up of head and neck squamous cell carcinoma (HNSCC) is associated to a survival benefit at 3 years.

**Clinical trial number:**

Not applicable (retrospective study).

## Introduction

Head and neck squamous cell carcinoma (HNSCC) accounts for more than 800,000 new cases annually worldwide, ranking sixth among the most common malignancies and causing more than 400,000 deaths [1, 2]. Despite therapeutic advances, patients with HNSCC still have a poor prognosis, mainly due to a high rate of locoregional recurrence (LR) within the first two years [3]. Regular and tailored follow-up is therefore essential [4, 5]. According to the French (SFORL) and American (NCCN) guidelines, conventional follow-up remains almost exclusively clinical [5, 6]. Currently, cervical imaging is recommended between 3 and 6 months after treatment to assess treatment efficacy, but no systematic imaging is recommended for follow-up, with the exception of annual chest CT. Performing cervical imaging during follow-up is optional, although tissue changes resulting from various therapeutic interventions may hinder clinical detection, especially of nodal recurrence [7, 8, 9]. Early detection of recurrence would improve overall survival, particularly by allowing salvage surgery [10, 11].

Recently, a cohort study of 902 HNSCC patients demonstrated a probable protective role of imaging-based follow-up in advanced stage patients (HR = 0.55), especially with 18 F-FDG PET/CT (HR = 0.29) [12]. A single center retrospective case-control study of 782 patients with 18 F-FDG PET/CT imaging showed similar results (OR = 0.71) [13]. Nevertheless, these conclusions lack external validity.

The main objective of this multicenter case-control study was to assess the impact on survival of a systematic post-treatment follow-up strategy using 18 F-FDG PET/CT compared with conventional follow-up in patients treated for HNSCC in two different French health areas.

## Materials and methods

### Population

Patients (≥ 18 years) diagnosed with histologically confirmed HNSCC between 1 January 2017 and 31 December 2020, treated with curative intent and showing a complete response on imaging at 3 to 6 months (M3-M6) in two health areas (area 1 and area 2) from two French regions were retrospectively included. Patients with residual disease after primary treatment were excluded (*n* = 224).

Several patient characteristics were collected at the time of the multidisciplinary team meeting, including history of cancer, history of other primary HNSCC, performance status index, smoking, alcohol consumption, primary tumour location, tumour stage according to the 8th edition of the American Joint Committee on Cancer (AJCC) classification [14], and presence of synchronous cancer.

The study was approved by the institutional ethics committee (29BRC22.0040). Patients consent to use of their data. The study complied with the General Data Protection Regulation (GDPR). We followed the STROBE guidelines (www.strobe-statement.org) for the design of this case-control study.

### Follow-up

All patients were followed up for at least 3 years within two different strategies: (i) conventional follow-up (CFU) according to usual recommendations [6], including clinical examination and nasofibroscopy every 2 months in the first year, every 3 months in the second year, and every 4 months in the third year + chest CT at M12, M24, and M36 (CFU group); (ii) CFU + systematic annual 18 F-FDG PET/CT instead of chest CT (PET/CT group), depending on clinicians usual practice.

### 18 F-FDG PET/CT imaging

The examinations were performed on Biograph-mCT systems (Siemens©, Erlangen, Germany) at the different centers, following guidelines from European Association of Nuclear Medicine [15]. After intravenous injection of 3–5 MBq/kg 18 F-FDG (Curium P©, Saclay, France), patients were asked to remain calm and at rest (lying down for approximately 1 h).

The CT scan was initially performed in the craniocaudal direction arms down with a whole-body protocol and injection of iodinated contrast agent (1.5 ml/kg) unless contraindication. Whole-body PET/CT data were acquired in 3D mode and included both emission images (2 to 3 min per step) and transmission images required for attenuation correction.

The PET emission images were corrected for background noise and random events, reconstructed with and without attenuation correction using the ordered subset expectation-maximisation iterative method (OSEM with point spread function (PSF) modelling and time-of-flight (TOF) acquisition capabilities) and smoothed with a Gaussian filter (full width at half maximum (FWHM) = 2 mm). The 40-slice Biograph scanner had a transverse field of view of 700 mm.

### Statistical analysis

Descriptive statistics were used to characterize the cohort at diagnosis. The incidence of primary metachronous cancers and of recurrences during follow-up were also analysed in both groups. Quantitative variables were described as median and range. Qualitative variables were expressed as number (n) and percentage (%). Fisher’s exact test and Mann-Whitney test were used to compare different variables between groups.

The primary endpoint was overall survival (OS), defined as the time from end of curative treatment to death, regardless of cause. Patients lost to follow-up were censored for survival analysis. The Kaplan-Meier method was used to estimate OS. The log-rank test was used to compare 3-year OS rates between the CFU and PET/CT groups in the entire cohort and stratified by AJCC stage or primary tumour location. Progression-free survival was defined as the time from end of curative treatment to recurrence or death. A Cox regression model was used to assess the effect of the addition of 18 F-FDG PET/CT on survival outcomes in our case-control study. This Cox model allowed analysis of time-related data, with survival as the main outcome of interest. By including 18 F-FDG PET/CT as an explanatory variable in the model, its association with survival was examined while adjusting for other relevant covariates such as age (years), sex (male), cancer history, performance status (0,1), smoking (> 10 pack-years), alcohol (> 3 drinks/day), primary site (oral cavity), and stage (early stage I/II), initial curative treatment (surgery) and end of treatment imaging (PET/CT). The assumptions of the Cox model were tested by graphical inspection of the Schoenfeld residuals over time.

The significance level for the p-value was set at 0.05. Statistical analyses were performed using SPSS v25 software (IBM Corp©, Armonk, NY).

## Results

### Population

Six hundred and ninety-seven patients were included, 508 in the CFU group and 189 in the PET/CT group. The clinical characteristics are shown in Table 1. The majority of patients were male (534/697 = 77%) with a median age of 62 years (range 26–95). Sixty-four (9%) had a history of HNSCC from another primary site. The most common tumour site was the oral cavity (261/697 = 37%). The preferred initial curative treatment was predominantly surgery (464/697 = 67%). One hundred and thirty-seven patients (20%), including 31 (16%) in the PET/CT group and 106 (21%) in the CFU group, were lost to follow-up after 3 years.

**Table 1:** Patient characteristics (*n* = 697)

	CFU Group		PET/CT group		Total		*p* value
Patients	508		189		697		
	n	%	n	%	n	%	
Gender (Male)	391	77	143	76	534	77	0.76
Age (years, median - standard deviation)	64	9	64	8	62	8	0.72
Cancer History	115	23	50	26	165	24	0.32
Prior HNSCC History	45	9	19	13	64	9	0.66
Smoking > 10 pack-years	405	80	173	92	578	83	**< 0.001**
Alcohol Consumption > 3drinks/day	211	42	115	61	326	47	**< 0.001**
PS							
0	351	69	115	61	466	67	**0.046**
1	142	28	59	31	201	29	0.4
2	15	3	12	6	27	4	**0.047**
3	0	0	3	2	3	0	**0.019**
Primary Site							
Oral Cavity	219	43	42	22	261	37	**< 0.001**
Oropharynx	118	23	68	36	186	27	**0.001**
HPV+	53	45	26	38	79	42	0.44
Larynx	121	24	46	24	167	24	0.92
Hypopharynx	42	8	24	13	66	9	0.08
CUP	8	2	9	5	17	2	**0.024**
AJCC Stage							
Early Stage (I/II)	196	39	65	34	261	37	0.33
I	103	20	41	22	144	21	0.68
II	93	18	24	13	117	17	0.09
Advanced Stage (III/IV)	312	61	124	66	436	63	0.33
III	98	19	25	13	123	18	0.07
IV	214	42	99	52	313	45	**0.017**
Synchronouse Cancer	73	14	38	20	111	16	0.08
Initial Curative Treatment							
Surgery	358	70	106	56	464	67	**< 0.001**
*Surgery Alone*	143	29	61	32	207	30	0.30
Positive Resection Margin	77	15	7	4	84	12	**< 0.001**
Radiotherapy	150	30	83	44	233	33	**< 0.001**
*Radiotherapy Alone*	25	5	32	17	57	8	**< 0.001**
Lost to Follow-Up	106	21	31	16	137	20	0.20

Abbreviations: HNSCC, Head and Neck Squamous Cell Carcinoma; PS, performance status; HPV, Human Papilloma Virus; CUP, Carcinoma of Unknown Primary; AJCC, American Joint Committee on Cancer

At the time of diagnosis, several patient characteristics were significantly less favorable in the PET/CT group than in the CFU group: fewer patients with performance status 0 (*n* = 115 (61%) vs. *n* = 351 (69%), *p* = 0. 046), higher rates of alcohol consumption (*n* = 115 (61%) vs. *n* = 211 (42%), *p* < 0.001) and tobacco use (*n* = 173 (92%) vs. *n* = 405 (80%), *p* < 0.001), and more advanced stages (AJCC IV) (*n* = 99 (52%) vs. *n* = 214 (42%), *p* = 0.017). In the CFU group, complete response on imaging (3 to 6 months after completion of treatment) was determined by MRI (*n* = 235), 18 F-FDG PET/CT (*n* = 162), or CT scan (*n* = 111). All patients in the PET/CT group were evaluated by 18 F-FDG PET/CT.

### Recurrences and metachronous primary cancers

The incidence of metachronous cancers within the 3-year follow-up period was similar between the CFU group (Lung (28), Other head and neck primary site (17), Oesophagus (5), Liver (4), Colorectal (4), Bladder (2), Prostate (2), Stomach, Lymphoma) and the PET/CT group (Lung (9), Prostate(2), Oesophagus (2), Breast, Liver, Pancreas, Stomach, neuroendocrine) (*n* = 64 (13%) vs. *n* = 18 (10%), *p* = 0.292). However, the number of recurrences was higher in the CFU group than in the PET/CT group (*n* = 116 (23%) vs. *n* = 29 (15%), *p* = 0.035). Metachronous cancers and recurrences were more frequently treated with curative intent in the PET/CT group than in the CFU group ((*n* = 36 (77%) vs. *n* = 99 (55%), *p* = 0.007). Of the 27 patients with subclinical recurrence, 4 had a second recurrence. The breakdown was as follows: 14 lymph node recurrences treated with surgery (12) and radiotherapy (2); 11 tumour recurrences treated with surgery (8), radiotherapy (2) and chemotherapy (1); and 6 metastases, 4 of which were treated with curative intent using pulmonary stereotactic radiotherapy (3) and liver surgery (1).

The heterogeneity of complete response on post-therapy imaging does not correlate significantly with recurrence. The observed recurrence rates were as follows: PET/CT: 80/351 (22.8%); MRI: 51/235 (21.7%); and CT: 20/111 (18.0%) *p* = 0.355.

### Survival

#### Overall analysis

**Fig. 1:**
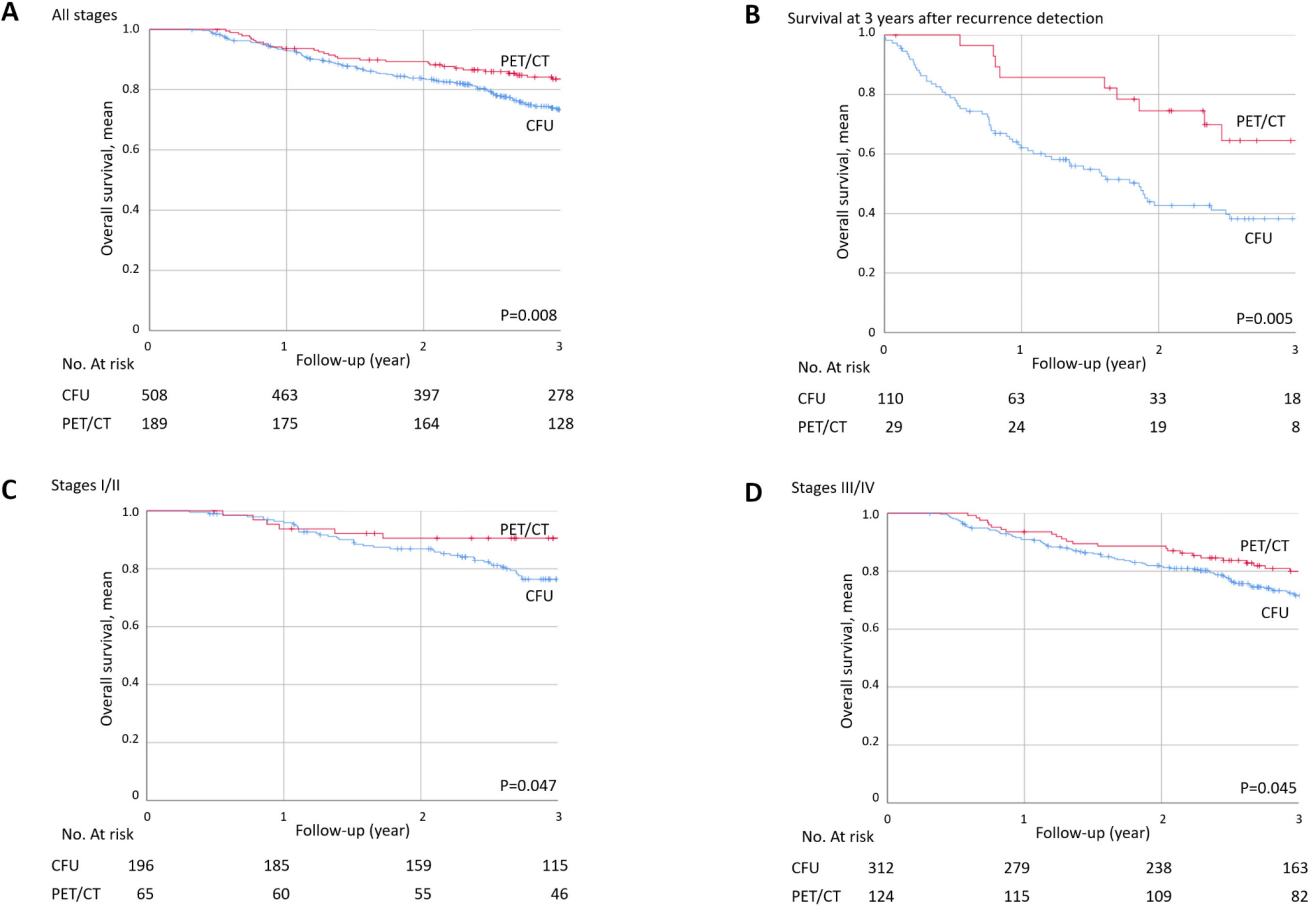
Three-year overall survival in PET/CT (*n* = 189) and CFU (508) groups (**A**) and 3-year overall survival after detection of recurrence in the PET/CT (*n* = 29) and CFU (*n* = 110) groups (**B**). Three-year OS in the PET/CT and CFU groups stratified by AJCC stage: stages III/IV (**C**), and I/II (**D**)

**Fig. 2:**
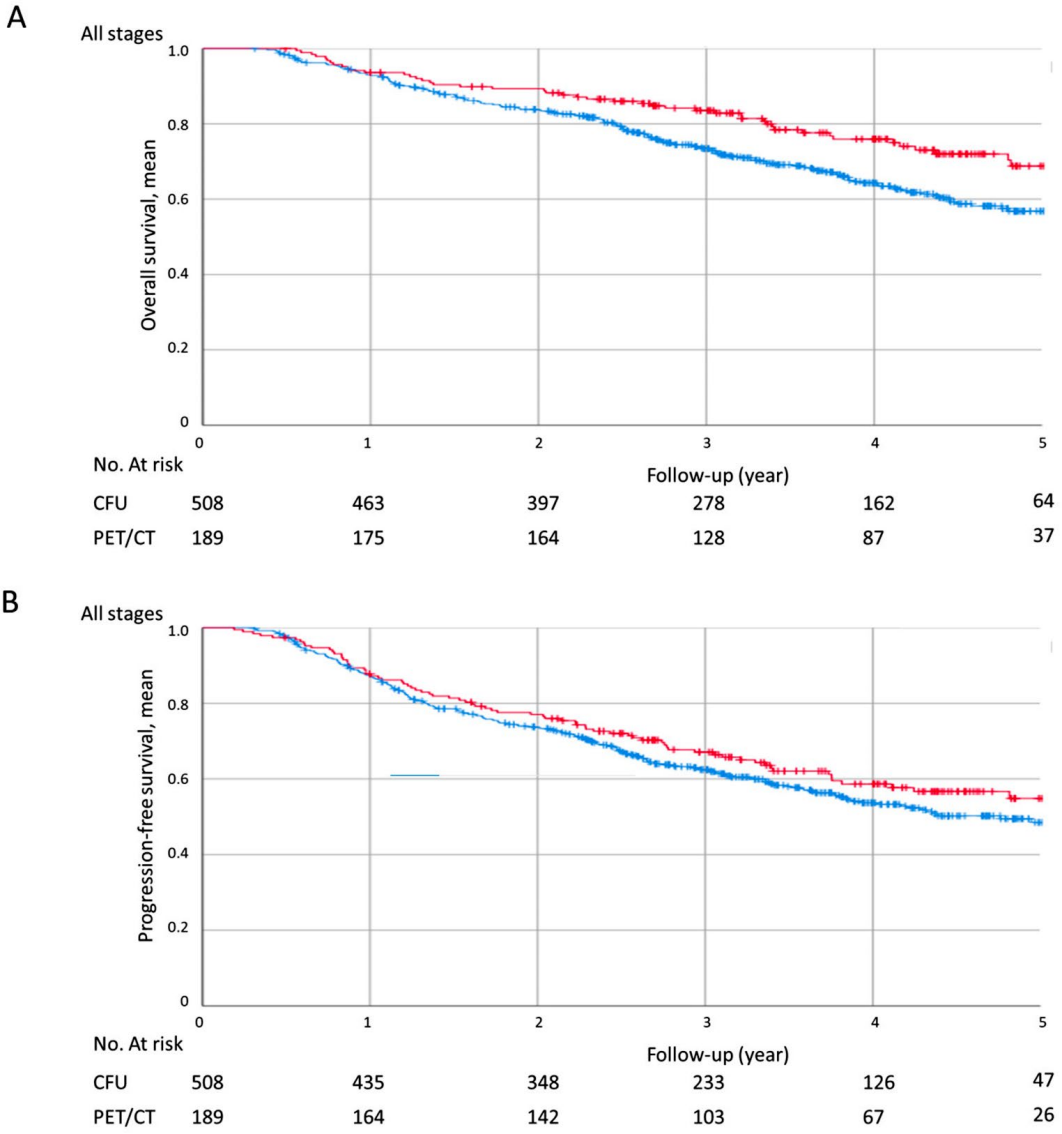
Five-year overall survival (**A**) and five-year progression-free survival (**B**) in PET/CT (*n* = 189) and CFU (508) groups

The 3-year overall survival (OS) was significantly better in the PET/CT group (*n* = 189) than in the CFU group (*n* = 508), regardless of the initial stage at diagnosis (83.5 ± 2.8% vs. 73.4 ± 2.1%, *p* = 0.008) (Fig. 1A). The 5-year OS was 64.7% ± 4.8% in the PET group versus 56.8% ± 2.8% in the CFU group (*p* = 0.007) (Fig. 2A). The 5-year progression-free survival was 52.6% ± 4.7% in the PET group versus 48.5% ± 2.8% in the control group (*p* = 0.194) (Fig. 2B).

Cox regression analysis showed a protective effect of 18 F-FDG PET/CT on survival during follow-up (HR = 0.56, 95%CI 0.397–0.795, *p* = 0.001) after adjustment for covariates (age, gender, oncological history, PS, smoking, alcohol consumption, stage, primary site, treatment and end of treatment imaging) (Table 2).

**Table 2:** Cox regression analysis with survival as the main outcome. In this analysis, PET/CT was included as an explanatory variable, while other parameters served as covariates

	*p* value	Odds ratio	IC 95%	
			Lower	Upper
PET/CT during follow-up	**0.001**	0.52	0.358	0.734
Covariates				
Gender (Male)	0.28	1.208	0.860	1.697
Age (years)	**0.001**	1.050	1.035	1.066
Cancer History	**0.037**	1.385	1.019	1.883
PS (0,1)	**0.001**	0.372	0.223	0.621
Smoking (> 10 pack-years)	0.053	1.506	0.995	2.283
Alcohol Consumption (> 3 drinks/day)	0.563	1.078	0.793	1.466
Stage (Early I/II)	0.081	0.758	0.556	1.034
Primary Location (Oral Cavity)				
Oropharynx	0.405	0.641	0.226	1.824
Larynx	0.856	0.905	0.310	2.646
Hypopharynx	0.771	1.1171	0.403	3.401
CUP	0.265	1.802	0.640	5.075
Initial Curative Treatment (Surgery)	**0.001**	0.492	0.357	0.678
End of treatment imaging (PET/CT)				
CT	**0.322**	1.286	0.782	2.114
MRI	**0.833**	0.939	0.521	1.691

Abbreviations: PS, performance status

The 3-year OS after recurrence was significantly better in the PET/CT group (*n* = 29) than in the CFU group (*n* = 110) (64.5 ± 9.8% vs. 38.2 ± 5.2%, *p* = 0.005) (Fig. 1B).

#### By AJCC stage

The 3-year OS for advanced stage III/IV was significantly better in the PET/CT group (*n* = 124) than in the CFU group (*n* = 312) (79.9 ± 3.7% vs. 71.5 ± 2.7%, *p* = 0.045) (Fig. 1C), as well as for early stage I/II disease (90.5 ± 3.7% vs.76.3 ± 3.2%, *p* = 0.047) (Fig. 1D).

#### By primary tumour location

**Fig. 3:**
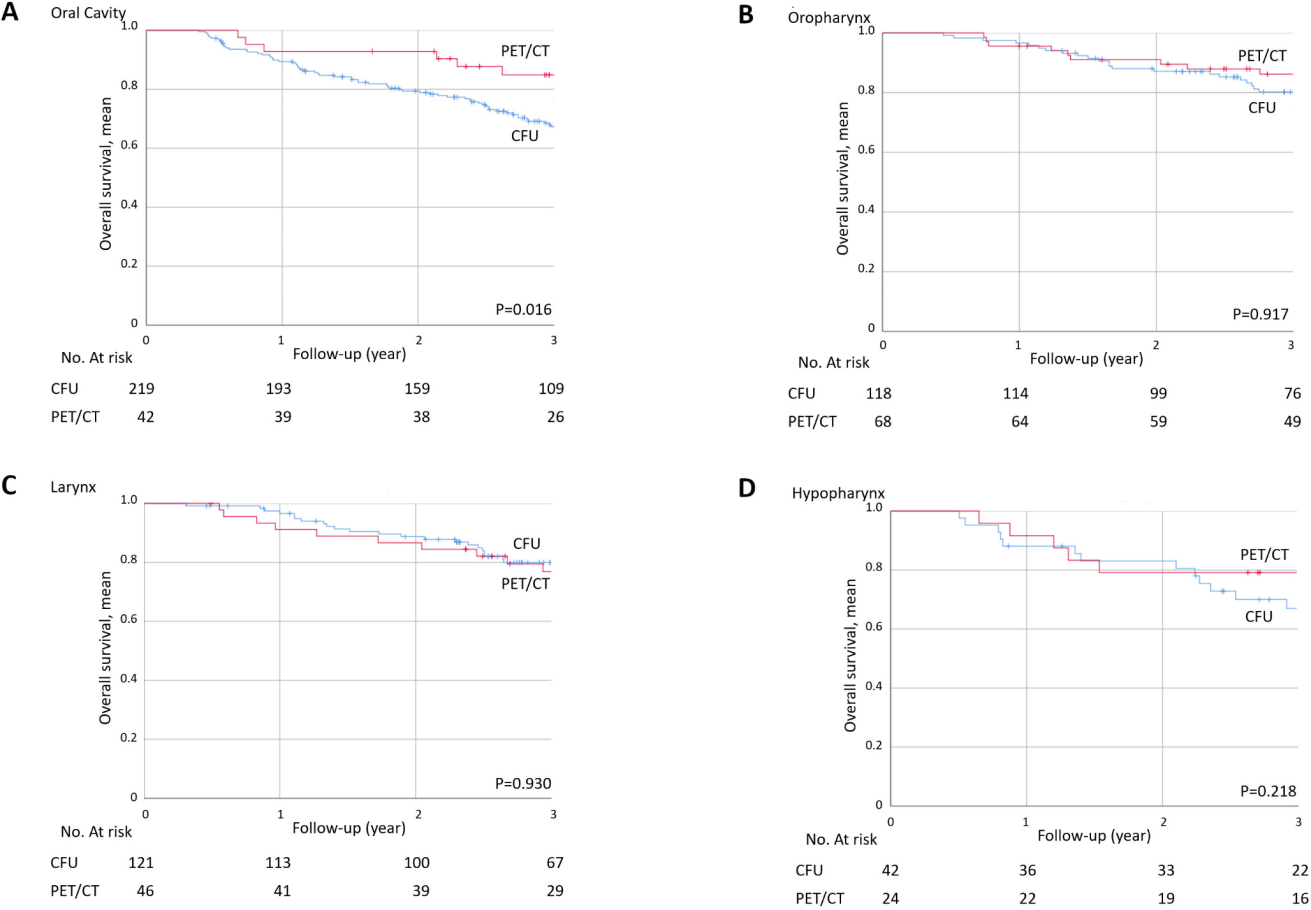
Three-year OS in the PET/CT (*n* = 42) and CFU (*n* = 219) groups for HNSCC of oral cavity (**A**), oropharynx (PET/CT (*n* = 68), CFU (*n* = 118)) (**B**), larynx (PET/CT (*n* = 46), CFU (*n* = 121)) (**C**), and hypopharynx (PET/CT (*n* = 24), CFU (*n* = 48)) (**D**)

The 3-year OS was significantly better for oral cavity tumours in the PET/CT group (*n* = 42) than in the CFU group (*n* = 219) (84.9 ± 5.7% vs. 67.3 ± 3.3%, *p* = 0.016) (Fig. 3A). There was no significant difference in 3-year OS between the PET/CT group and the CFU group for oropharyngeal (86.2 ± 4.3% vs. 80.2 ± 3.8%, *p* = 0.917) (Fig. 3B), laryngeal (76.6 ± 6.5% vs. 80 ± 3.8%, *p* = 0.930) (Fig. 3C) and hypopharyngeal tumours (79.2 ± 8.3% vs. 67 ± 7.6%, *p* = 0.218) (Fig. 3D).

#### Additional analyses

The 3-year OS analysis comparing the two groups based on prior HNSCC history, performance status, smoking, alcohol consumption, initial treatment, and positive resection margins is shown in Table 3.

**Table 3:** Three-year OS analysis comparing CFU and PET group by prior HNSCC history, performance status, smoking > 10 pack-year, alcohol consumption > 3drinks/day, initial treatment, and positive resection margin

		*N*	3 y OS CFU		3 y OS PET		*p* value
			%	σ	%	σ	
Prior HNSCC history	No	532	72.4	2.2	84.5	2.8	**0.007**
	Yes	165	81.4	5.6	71.5	12.2	0.922
PS	0	466	78.2	2.4	87.3	3.1	0.088
	1	201	66.3	4.1	78.0	5.9	0.071
Smoking > 10 pack-year	No	119	74.6	4.5	87.1	8.6	0.519
	Yes	578	73.1	2.3	83.2	2.9	**0.006**
Alcohol Consumption > 3 drinks/day	No	371	74.6	2.7	83.1	3.3	**0.021**
	Yes	326	71.8	3.3	83.9	3.5	0.104
Initial treatment	Surgery	464	76.9	2.3	90.4	2.9	**0.001**
	Radiotherapy	233	65.0	4.1	73.7	5.1	0.457
Positive resection margin	No	380	76.9	2.6	91.7	3.0	**0.004**
	Yes	84	75.9	5.3	80.0	10.3	0.196

Abbreviations: HNSCC, Upper Aerodigestive TractHead and Neck Squamous Cell Carcinoma; PS, Performance status

## Discussion

Our study showed that patients with complete response after treatment for head and neck squamous cell carcinoma (HNSCC) who underwent additional systematic annual 18 F-FDG PET/CT to conventional follow-up had a better 3-year OS than the others (83.5 ± 2.8% in PET/CT group vs. 73.4 ± 2.1% in CFU group, *p* = 0.008). 18 F-FDG PET/CT during follow-up was protective after adjustment for other covariates (OR = 0.56, 95%CI: 0.397–0.795, *p* = 0.001). These results are consistent with those of two recent large single center studies. Indeed, the cohort study by Anzai et al. including 902 patients showed a significant lower mortality in HNSCC patients with follow-up by 18 F-FDG PET/CT or conventional imaging (HR = 0.29, 95%CI 0.09–0.94, *p* = 0.04) [12]. Similarly, the case-control study by Leclère et al. in 782 patients reported better survival with 18 F-FDG PET/CT follow-up (OR = 0.71, 95%CI 0.57–0.88, *p* = 0.002) [13]. However, a retrospective study of 257 HNSCC patients showed no difference in survival according to the method of recurrence detection, either by 18 F-FDG PET/CT or clinically, with reported 3-year disease-free survival of 41% vs. 46% (*p* = 0.91) and 3-year OS of 60% vs. 54% (*p* = 0.70), respectively [16].

We found that 3-year OS was significantly better in the PET/CT group than in the CFU group (64.5 ± 9.8% vs. 38.6 ± 5.2%, *p* = 0.005) after detection of recurrence with each strategy. We hypothesize that this survival difference in our study is related to the overall detection rate of 15% (29/189) for subclinical recurrence in the PET/CT group, a rate similar to the 14% reported in the meta-analysis of 7 studies with a total of 907 patients by Sheikhbahaei et al. [17]. By detecting 18 metachronous primary cancers (MPC) with 18 F-FDG PET/CT, our overall rate of subclinical events was 25% (47/189). This rate is consistent with the findings of a retrospective study by Dunsky et al., who reported 24 (20%) asymptomatic lesions (either recurrent disease or new primary) detected by 18 F-FDG PET/CT in 123 asymptomatic patients treated for HNSCC [18].

In a further subgroup analysis, patients diagnosed at an advanced stage had a better 3-year survival when followed-up with CFU + 18 F-FDG PET/CT (79.9 ± 3.7% vs. 71.5 ± 2.7%, *p* = 0.045). Several series have shown that advanced AJCC stages III-IV have a higher risk of asymptomatic recurrence and therefore recent published guidelines recommend 18 F-FDG PET/CT for surveillance of these patients [9, 13, 17]. More surprising is the existence of a survival difference in patients diagnosed at an early stage (90.5 ± 3.7% vs. 76.3 ± 3.2%, *p* = 0.047). To our knowledge, no other study has shown such a survival benefit from the use of 18 F-FDG PET/CT in early stage (I/II) follow-up, which is an interesting direction for future research.

Regarding primary tumour location, our results highlighted a better 3-year survival in the PET/CT group for oral cavity cancers (84.9 ± 5.7% vs. 67.3 ± 3.3%, *p* = 0.016). For other primary location, the lack of difference may probably be explained by insufficient statistical power in different subgroups, especially for oropharynx and hypopharynx. In addition, although the difference is not significant, there were fewer HPV + patients in the PET/CT group (38%) than in the CFU group (45%). In the study by Leclère et al., the oropharynx was the most common primary site (*n* = 176/497) of the cohort and also the only site with significantly better 3-year survival in patients followed by 18 F-FDG PET/CT (69.9% vs. 60.5%; *p* = 0.04) [13]. There seems to be less interest in survival in laryngeal cancer, probably due to the lower lymphophilia in this area.

10% of metachronous primary cancers (MPC) were detected in our PET/CT group (*n* = 18), half of which were in the lung region (*n* = 9, 50%). These results are consistent with the literature, as the reported overall incidence of MPC in patients treated for HNSCC ranged from 8.1 to 16.3% depending on the series [13, 19, 20], and the annual rate remained constant during the follow-up period [21]. Furthermore, Liu et al. highlighted that the main sites of MPC were the head and neck region, oesophagus and lung in a cohort of 5,914 patients treated for pharyngo-laryngeal cancer [22]. In a meta-analysis of 26 studies, Hoxhaj et al. found similar results for MPC sites in 72,450 patients, mainly due to the same risk factors (alcohol and tobacco use) [23]. Regarding studies focusing on the diagnostic performance of 18 F-FDG PET/CT, Krabbe et al. reported a rate of 6.7% of lung MPC in a cohort of 149 asymptomatic HNSCC patients, compared to 4.7% (9/189) in our study [24]. Furthermore, in our previous prospective series, we reported an overall MPC detection rate of 5.1% (6/116) and 7.7% (7/91) using 18 F-FDG PET/CT in clinically asymptomatic patients at 6 months and 1 year post-treatment, respectively [25, 26]. This small difference could be explained by the difficulty in differentiating a solitary lung metastasis from a metachronous disease in the case of squamous cell carcinoma histology.

Finally, recurrences and MPC were more frequently treated with curative intent in the PET/CT group than in the CFU group (76.6% vs. 55%, *p* = 0.007), confirming our hypothesis that earlier detection of events by 18 F-FDG PET/CT improves patient management.

For other cancers, such as oesophageal cancer, systematic follow-up imaging is recommended. Recent evidence, including from the 4,682-patient ENSURE trial (NCT03461341) [27], suggests that intensive surveillance with annual thoraco-abdominal CT scans for three years improves overall survival in patients who have undergone primary surgery. Similarly, in our study, 3-year OS of patients who underwent initial surgical treatment in the PET group was better compared to those in the CFU group (90.4% vs. 76.9%, *p* = 0.001). In contrast, 3-year OS of patients initially treated with radiotherapy was similar between PET and CFU groups (73.7% vs. 65.0%, *p* = 0.457), consistent with the findings of Chen et al. [28]. This is likely explained by the technical challenges associated with performing curative-intent surgery in cases of recurrence in patients who were not initially treated with surgery, even when the recurrence is subclinical. For lung cancer, chest CT scans are usually performed every six months for three years. Notably, PET/CT is recommended for the assessment of recurrence in both scenarios.

Our study has several limitations. First, we selected patients with complete response at 3 months in the inclusion criteria to focus on asymptomatic patients. In the absence of specific recommendations, the imaging modalities used for the 3-month evaluation varied, and patients followed with 18 F-FDG PET/CT were preferentially evaluated with this modality. However, recent study results tend to show that 18 F-FDG PET/CT would be the most effective in this indication [26, 29], in particular with an excellent negative predictive value. This may partly explain the higher incidence of recurrence in our conventional follow-up (CFU) group. Second, it is also noteworthy that patients in the CFU group were more likely to undergo surgery (70% vs. 56%, *p* < 0.001) and had more microscopically involved margins (15% vs. 4%, *p* < 0.001). Despite this, patients monitored with 18 F-FDG PET/CT were more likely to be treated with curative intent when a recurrence or a MPC was detected, due to earlier detection. Third, the proportion of patients receiving conventional follow-up was higher, reflecting compliance with current recommendations. The choice of follow-up modality was determined by clinicians’ choice, probably depending on their length of practice, as PET/CT remains a relatively recent innovation. Finally, the follow-up period was limited to 3 years. The optimal schedule of 18 F-FDG PET/CT use and the duration of follow-up remain to be determined.

As future perspective, some studies have evaluated the usefulness of 18 F-FDG PET/MRI in post-treatment follow-up [30]. A prospective study of 82 HNSCC patients who underwent regular 18 F-FDG PET/CT followed by 18 F-FDG PET/MRI during follow-up showed that these scans had similar performance in detecting distant recurrence and MPC (*p* = 0.919) [31]. This suggests that the metabolic nature of the imaging is critical for detecting recurrence in areas altered by surgery and/or radiotherapy.

## Conclusion

The use of 18 F-FDG PET/CT as an alternative to annual chest CT in the follow-up of head and neck squamous cell carcinoma (HNSCC) is associated to a survival benefit at 3 years, regardless of stage, particularly for oral cavity cancers.

## Data Availability

The datasets generated during and/or analysed during the current study are available from the corresponding author on reasonable request.
